# Nasal Administration of Anti-CD3 Monoclonal Antibody (Foralumab) Reduces Lung Inflammation and Blood Inflammatory Biomarkers in Mild to Moderate COVID-19 Patients: A Pilot Study

**DOI:** 10.3389/fimmu.2021.709861

**Published:** 2021-08-12

**Authors:** Thais G. Moreira, Kimble T. F. Matos, Giovana S. De Paula, Thais M. M. Santana, Raquel G. Da Mata, Fernando C. Pansera, Andre S. Cortina, Marcelle G. Spinola, Clare M. Baecher-Allan, Gerson D. Keppeke, Jules Jacob, Vaseem Palejwala, Karen Chen, Saef Izzy, Brian C. Healey, Rafael M. Rezende, Rogerio A. Dedivitis, Kunwar Shailubhai, Howard L. Weiner

**Affiliations:** ^1^ Ann Romney Center for Neurologic Diseases, Department of Neurology, Brigham and Women’s Hospital, Harvard Medical School, Boston, MA, United States; ^2^ Escola Paulista de Medicina, Universidade Federal de São Paulo, São Paulo, Brazil; ^3^ Santa Casa de Misericordia de Santos, Santos, Brazil; ^4^ Tiziana LifeScience, Doylestown, PA, United States; ^5^ Department of Radiology, Boston Children’s Hospital, Harvard Medical School, Boston, MA, United States

**Keywords:** foralumab, anti-CD3, COVID-19, SARS-CoV-2, immune responses

## Abstract

**Background:**

Immune hyperactivity is an important contributing factor to the morbidity and mortality of COVID-19 infection. Nasal administration of anti-CD3 monoclonal antibody downregulates hyperactive immune responses in animal models of autoimmunity through its immunomodulatory properties. We performed a randomized pilot study of fully-human nasal anti-CD3 (Foralumab) in patients with mild to moderate COVID-19 to determine if its immunomodulatory properties had ameliorating effects on disease.

**Methods:**

Thirty-nine outpatients with mild to moderate COVID-19 were recruited at Santa Casa de Misericordia de Santos in Sao Paulo State, Brazil. Patients were randomized to three cohorts: 1) Control, no Foralumab (n=16); 2) Nasal Foralumab (100ug/day) given for 10 consecutive days with 6 mg dexamethasone given on days 1-3 (n=11); and 3) Nasal Foralumab alone (100ug/day) given for 10 consecutive days (n=12). Patients continued standard of care medication.

**Results:**

We observed reduction of serum IL-6 and C-reactive protein in Foralumab alone *vs*. untreated or Foralumab/Dexa treated patients. More rapid clearance of lung infiltrates as measured by chest CT was observed in Foralumab and Foralumab/Dexa treated subjects *vs*. those that did not receive Foralumab. Foralumab treatment was well-tolerated with no severe adverse events.

**Conclusions:**

This pilot study suggests that nasal Foralumab is well tolerated and may be of benefit in treatment of immune hyperactivity and lung involvement in COVID-19 disease and that further studies are warranted.

## Introduction

The new beta-coronavirus Severe Acute Respiratory Syndrome Coronavirus 2 (SARS- CoV-2) has infected over 200 million people world-wild and represents the greatest global public health crises since the pandemic influenza outbreak of 1918 (1, 2). Clinical trials have focused on anti-viral therapy (3–5) and treatment to modulate the immune system including corticosteroids (6, 7), convalescent plasma (8, 9), immunoglobulins (10, 11) and tocilizumab (12–14).

SARS-CoV-2 infection involves prominent immune changes including infiltration of monocytes to the lungs associated with elevated production of pro-inflammatory cytokines known as cytokine storm (15–17). The COVID-19 hyperinflammatory syndrome can led to multiorgan failure and acute respiratory distress syndrome that represents the leading cause of mortality in COVID-19 (17). Increased cytotoxic follicular helper cells (TFH) and cytotoxic T helper cells and a decrease in SARS-CoV-2-reactive Tregs were observed in hospitalized COVID-19 patients. A strong cytotoxic TFH response was observed early in the illness (18). A reduction of Tregs could be an important contributing factor to the hyperactive immune system and lung damage in COVID-19 patients. This is consistent with animal studies in which Treg depletion led to acute encephalitis and increased mortality in mice infected with murine coronavirus (19).

Considering that an excessive immune response plays an important role in COVID-19 infection, we hypothesized that enhancing T regulatory cells (Treg) could be of benefit in SARS-CoV-2 infection (20–22). Tregs play a key role in maintaining tolerance to self-antigens and in suppression of excessive immune responses in autoimmune conditions (23, 24). We have been investigating mucosal administration of anti-CD3 monoclonal antibody (mAb) as a novel approach to induce Tregs and suppress inflammation in models of autoimmunity (25–28). We have shown that nasal anti-CD3 induces IL-10 dependent Tregs that suppress inflammation and disease progression in several inflammatory diseases models including experimental autoimmune encephalomyelitis, lupus and arthritis (26, 29).

We are developing nasal Foralumab, a fully human anti-CD3 monoclonal antibody (mAb), for treatment of secondary progressive multiple sclerosis (SPMS). In preparation for a clinical trial of nasal Foralumab in SPMS we conducted a dose-ranging safety trial in healthy volunteers at the Brigham and Women’s Hospital. Foralumab had previously been tested in subjects with colitis given intravenously (30). We found that nasal Foralumab was safe and was immunologically active as measured by suppression of CD8+ T cell responses and induction of CD4+ IL-10 responses (unpublished). The characterization of the immune response to nasal anti-CD3 in healthy volunteers is ongoing. Given the crises caused by the COVID-19 pandemic and the immediate need for novel approaches to treat the illness, we elected to perform a pilot trial of nasal Foralumab in COVD-19 subjects since we had found that Foralumab was safe in healthy individuals and had immunomodulatory effects.

We chose to treat mild to moderate COVID-19 subjects who were outpatients seen at the Santa Casa de Misericordia de Santos Hospital in Brazil. A dose of 100ug/day for 10 consecutives days was selected based on our experience with nasal Foralumab in healthy individuals. We randomized subjects to receive no treatment *vs*. treatment with Foralumab/Dexa *vs*. treatment with Foralumab alone. The measurement of inflammatory blood markers IL-6, CRP and D-dimer was used as the primary outcome.

## Methods

### Patient Recruitment and Trial Design

Patients with flu-like symptoms consistent with COVID-19 infection were evaluated at Santa Casa de Misericordia de Santos Hospital emergency ward and screened for the study. Inclusion criteria comprised 1) a positive RT-PCR COVID test; and 2) mild to moderate disease with an oxygen saturation over 93% and no requirement for oxygen. Exclusion criteria included (1) infectious disease: syphilis, hepatitis and HIV; (2) Pregnancy; (3) less than 18 years old; (4) chronic kidney disease; (5) cancer or other immunodeficiencies; and (6) elevated glycated hemoglobin for patients with diabetes. Of 60 patients screened for the study, 39 patients were enrolled. 10 were negative for COVID by PCR and 11 elected not to participate after screening.

Patients were randomized into three cohorts: no Foralumab treatment, nasal Foralumab/Dexa, and nasal Foralumab alone. Treatment was administered in an open label fashion. Patients randomized to Foralumab treatment were visited by a nurse for daily drug administration. 50μg of Foralumab was administered by nose drop to each nostril (total 100μg). Patients receiving dexamethasone received 6 mg of oral dexamethasone on days 1-3. Patients in the control group did not receive foralumab. All arms were allowed to continue background antibiotics. Corticosteroids were not given per protocol but patients were able to access them through self or other healthcare provider outside the hospital.

**Figure 1:**
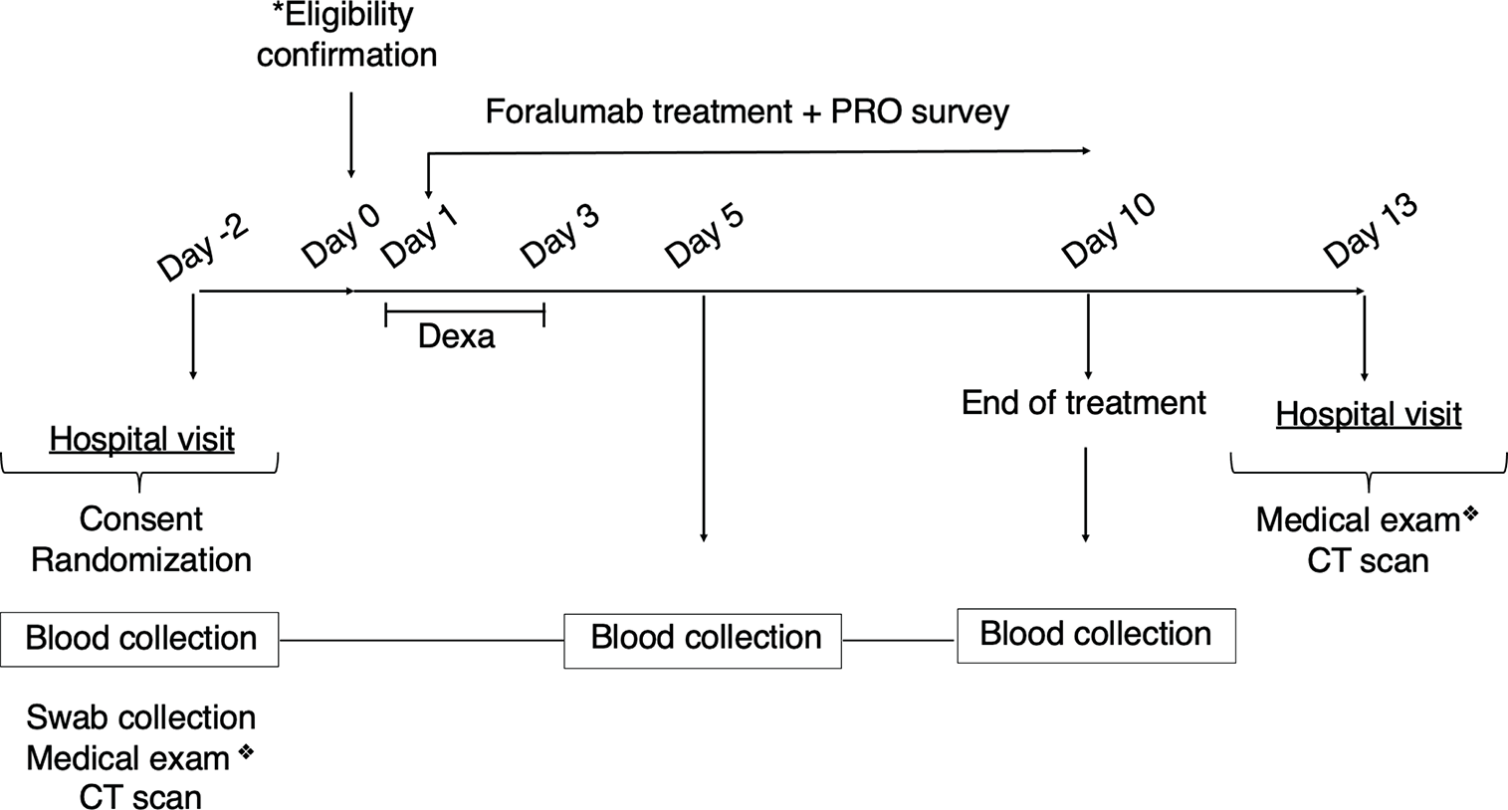
Clinical Study Design. Symptomatic patients were consented and randomized on day -2. Blood and nasopharyngeal swab collection, CT scans and medical exam was also performed at day -2. *Eligibility was confirmed on day 0 (SARS-CoV-2 PCR positive results, infectious disease negative results). Foralumab treatment started at day 1 for 10 days. Dexamethasone (6mg/day) was given on days 1-3 to Foralumab/Dexa group. PRO data was collected daily during treatment (day 1 to day 10) and symptomatology was accessed during medical exam at the beginning and completion of study (day -2 and day 13). Blood collection for biomarkers follow-up was performed at day 5 and day10. Patient returned to the hospital for clinical exam and CT scan follow up on day 13. ❖ Symptomatology and drug use assessment. CT, computerized tomography; PRO, patient reported outcome.

Foralumab treatment was given for 10 consecutive days. Patients returned to hospital at day 13 for clinical exam and lung CT scan follow up. All patients had a lung CT scan prior to entering the study (**Figure 1**).

Patients completed a daily clinical outcome questionnaire to assess 15 common COVID-19 related symptoms designed based on FDA guidelines (see below). This scoring system generated a numeric value for each day. Overall wellness was accessed using Baker Wong scale for pain assessment. Patients allocated to the control group did not receive placebo and thus were not visited by the clinical team apart from blood collection on days 5 and 10. For these patients, the daily survey was conducted by phone.

### Study Approval

The study was conducted according to the Brazilian regulatory agency for clinical research and good clinical practice guidelines and declaration of Helsinki. Ethical committee approval was granted by Universidade Metropolitana de Santos – UNIMES (CAAE: 38056120.1.0000.5509). The investigators designed the trial, collected the data, and performed the analysis. In addition to providing Foralumab, Tiziana Life Sciences also provided financial assistance to the trial but did not participate in statistical analysis or data interpretation.

### Laboratory Tests

Nasopharyngeal swabs were used to screen for COVID-19 by RT-PCR. Clinical laboratory tests were performed at S’agapo Laboratory and included: complete blood counts, IL-6, D-dimer, CRP, COVID-19 Serology, HIV, syphilis, pregnancy, hepatitis, and glycated hemoglobin. White blood counts were measured by flow cytometry and impedance. CRP and glycated hemoglobin were measured by turbidimetry, D-dimer was measured by immunoturbidimetry, and IL-6 was measured by chemiluminescence. IL-18 was measured using ProcartaPlex multiplex assay (Thermo Fisher, EPX450-12171-901).

### Lung CT and Analysis

Lung CT scan was performed at Santa Casa de Misericordia de Santos Hospital using a 16 channel (Toshiba-Alexion) CT scanner. Contrast was not used. Scan coverage was from apex of the lung to the level of bilateral adrenals. Tube voltage was between 100-120Kv. Parenchyma slide thickness was 1mm.

Lung injury consisted of patchy shadowing and ground glass appearance which was graded on a scale of 0 to 4 as follows: 0 = no detectable abnormalities or lung involvement <5%; Stage 1 = mild lung involvement involving approximately 10% of lung area, Stage 2= moderate lung involvement with patchy shadowing and ground glass lesions involving approximately 25% of lung area, Stage 3 = severe confluent ground glass lesions and consolidation involving 25% to 50% of lung area; Stage 4 very severe ground glass lesions and consolidation involving more than half of the lung area.

Baseline lung CT scans obtained on day -2 were compared to scans obtained on day 13. Patients were classified as worsened if they increased by one or more stage, improved if they decreased by one stage and as having marked improvement if they decreased by 2 or more stages. Patients were stable if they did not change stages. Lung CT analysis was performed by three radiologists in a blinded fashion.

### Patient Reported Outcomes (PRO) and Medical Report Outcome

Patient reported outcomes were accessed daily using a symptomatology survey based on FDA guidelines (Assessing COVID-19-related symptoms in Outpatient Adult and Adolescent Subjects in Clinical Trials of Drug and Biological Products for COVID-19 Prevention or Treatment) (https://www.fda.gov/media/142143/download). PRO consisted of 15 questions with the following response system: (1) Anosmia (loss of smell): 0=normal, 3=reduced, 5=completely lost). (2) Dysgeusia (loss of taste): 0=normal, 3=reduced, 5=completely lost). (3) Cough: 0=not present, 3 =present some time, 5=present more than half the day. (4) Headache: 0=not present, 3=present some time, 5=present more than half the day); (5) Throat ache: 0=not present, 3=moderate, hurts when swallowing, 5=strong, intense pain when swallowing. (6) Dyspnea: 0=not present, 3= moderate, some lack of air, 5=strong, difficult breathing. (7) Nausea/Vomiting: 0=not present, 3= nausea without vomiting, 5=vomiting. 8) O2 Saturation: 0= > 95, 3 = 94-95%, 5 = 91-93%. (9) Diarrhea was evaluated according to the Bristol Scale (ref) 0=type 0-4, 3=type 5 or 6, 5=type 7. (10) Rhinorrhea: 0=not present, 3=nose with mucus, 5=runny nose (liquid)); (11) Abdominal pain: 0=not present, 3=moderate, 5=intense). (12) Myalgia: 0=not present, 3=moderate, 5=intense (full body). (13) Fever: 0=not present, 3 = 37-38C, 5= >38.0C. (14) Conjunctivitis: 0=absent, 5=present. (15) Appetite: 0=normal, 3=reduced, 5=completely lost. General well-being (how you are feeling today) was assessed using the Baker Wong scale for pain assessment (0-10). The maximum possible score was 85.

We also accessed COVID-19 symptoms reported at day -2 as compared to symptomatology at day 13. For this, we stratified patient reported symptoms according to Domains as follows: Domain 1 (weakness, fatigue, inappetence, body ache, backpain); Domain 2 (fever, chills, sweating); Domain 3 (nausea, diarrhea, epigastric pain); Domain 4 (ageusia); Domain 5 (anosmia); Domain 6 (runny nose, odynophagia, sneezing); Domain 7 (headache, anxiety, eye pain, dizziness); Domain 8 (cough, dyspnea, chest pain). Patient was scored for one symptom in each domain.

### Statistical Analysis

The demographic characteristics of the three treatment groups were summarized using means/standard deviations for continuous outcomes and number/percentages for dichotomous outcomes. The patient reported outcomes were measured daily for 10 days, and the change with time in each treatment group was estimated using a linear mixed model with a random intercept and slope. The fixed effects in the model were two indicators for the Foralumab groups, day, and two day by treatment group interaction terms. The estimated slope in each treatment group, and the pairwise group comparison of the slopes are reported. For the presence of symptoms at each domain, the number/percentage for each domain was calculated prior to starting the treatment and at day 10, and the percentage was compared between the treatment groups using a chi-squared test. The total number of domains at each time point was compared across the treatment groups using a Kruskal-Wallis test. For biomarkers, we used linear mixed effects regression model with a random intercept to compare the three groups in terms of change from baseline (day -2) at day 5 and day 10. The primary analysis was the three-group comparison, and this was completed using appropriate contrasts from the linear mixed model. Comparisson between the treatment groups for lung injury (Lung CT scan) was calculated using a chi-squared test. A p value of < 0.05 was considered statistically significant. All analyses were performed with R software and graph prism was used for graph representation.

## Results

### Patients

The baseline demographics are shown in **Table 1**. Thirty-nine patients participated in the study, 64.1% females (n=25) and 35.9% males (n=14). The mean age of subjects was 41.1 ± 14.2. with control subjects 33.5, Foralumab/Dexa subjects 41, and Foralumab subjects 44.5. The majority of patients were white. Twenty-three (41%) patients had one or more comorbidities with the most common being obesity. Obesity (BMI>30 Kg/m^2^) occurred in 37.8% of patients and was observed in all groups (31). Grade 2 obesity was observed in one control subject and one subject in the Foralumab group. Eleven patients (28.2%) reported the use of corticosteroids (prednisolone or dexamethasone) prior to entry in the study. Patients that did not use corticosteroids prior to the study were randomized to the control or the Foralumab group. Patients that used corticosteroids prior to the study were randomized to the control or Foralumab/Dexa group.

**Table 1:** Patient Characteristics.

Characteristic	Control	Foralumab/Dexa	Foralumab	Total
	(n = 16)	(n = 11)	(n = 12)	(n = 39)
**Gender - no. (%)**				
Female	10 (62.5)	6 (54.5)	9 (75)	25 (64.1)
Male	6 (37.5)	5 (45.5)	3 (25)	14 (35.9)
**Age ^#^( ± SD)**	33.5 ( ± 18.19)	41 ( ± 9.14)	44.5 ( ± 11)	41.1( ± 14.2)
^†^ **Race - no. (%)**				
White	13 (81.3)	9 (81.8)	10 (83.3)	32 (82)
^††^Not white/black	2 (12.5)	2 (18.2)	2 (16.7)	6 (15.4)
Black	1 (6.25)	0	0	1 (2.6)
**Comorbidities - no. (%)**				
Hypertension	3 (18.8)	0	4 (33.3)	7 (17.9)
Diabetes	2 (12.5)	0	2 (16.7)	4 (10.3)
Cardiovascular disease	2 (12.5)	0	3 (25)	5 (12.8)
Obesity ^❖^BMI > 30	3 (21.4)^*^	5 (45.5)	6 (50)	14 (37.8)^*^
**Drug use prior to study - no. (%)**				
Corticosteroids	6 (37.5)	5 (45.5)	0	11(28.2)
Azithromycin	5 (31.3)	4 (36.4)	3 (25)	12 (30.8)
Ivermectin	1 (6.25)	0	3 (25)	4 (10.3)
Hydroxychloroquine	1 (6.25)	0	1 (8.3)	2 (5.12)
**Drug use during the study - no. (%)**				
Corticosteroids^‡^	5 (31.3)	11(100%)	0	21 (53.8)
Azytromicin	9 (56.3)	5 (45.5)	9 (75)	23 (59)
Ivermectin	0	0	2 (16.7)	2 (5.12)
Acetylcysteine	3 (18.75)	1 (9)	1 (8.3)	5 (12.8)
**Anti-COVID-19 antibodies - no. (%)**				
IgM (day -2)	4 (25)	3 (27.3)	2 (16.6)	9 (23.1)
IgG (day -2)	1 (6.25)	2 (18.2)	3 (25)	6 (15.4)
IgM (day 10)	11(68.8)	6 (54.5)	10 (83.3)	27 (69.2)
IgG (day 10)	13 (81.3)	9 (81.8)	10 (83.3)	32 (82)

**^#^** Plus–minus values are means ± SD. ^†^Race was reported by the patients as well as weight and height.

^††^Ethnic and skin mixed color category named “Pardo” used by the Brazilian Institute of Geography and Statistics (IBGE). ^❖^The body-mass index (BMI) is the weight in kilograms divided by the square of the height in meters. *Two patients in the control group could not report weight and height (total n=14). Drug use was reported by patients to physicians at day-2 and day 13. ^‡^Background Immunoglobulin (Ig).

Drug use during the study: Twenty-three patients (58.9%) received azithromycin prescribed their physicians. Two patients took the anti-helminth drug ivermectin and 5 patients took the drug acetylcysteine. At the 13day follow-up visit, 5 patients in control group reported taking up to 5 days of off label steroids during the study.

IgM and IgG anti-COVID antibodies were measured on day -2 and day 10 of the study. Nine subjects (23.1%) had anti-COVID IgM antibodies on day -2 which increased to 27 subjects (69.2%) at day 10. Six subjects (15.4%) had anti-COVID IgG antibodies on day -2 which increased to 32 subjects (82%) at day 10. No differences in the development of IgM or IgG antibodies were observed between the groups.

### Patient Reported Outcomes (PRO)

Patients completed a daily clinical outcome questionary to assess 15 common COVID-19 related symptoms. There were no differences in patient reported outcome among groups (**Table 2**).

**Table 2:** Estimated change per day in patient reported outcomes (PRO).

	Anosmia	Dysgeusia	Baker Scale	Total Score
Control (n=16)	-0.25	-0.18	-1.39	-1.3
Foralumab/Dexa (n=11)	-0.18	-0.24	-1.31	-1.23
Foralumab (n=12)	-0.15	-0.19	-1.23	-1.17
Foralumab/Dexa – control	0.35	0.48	0.82	0.83
Foralumab – control	0.17	0.93	0.64	0.67
Foralumab - Foralumab/Dexa	0.72	0.57	0.83	0.85

The change per day in each group was estimated using a linear mixed model with a random intercept and slope. To compare groups, the estimated difference in the change per day is reported with p-value.

### COVID Symptomatology

We compared patient symptomatology at day -2 *vs*. day 13 according to the 8 Domains (eg., fever, gastrointestinal symptoms, and respiratory symptoms) described in methods above. At day -2, patients had been experiencing symptoms for an average of 6 days in an average of 5 Domains. As shown in **Table 3**, most patients improved during the course of the study with no major differences between the treatment groups. At the end of the study 23 of 39 patients (58.9%) were asymptomatic; 8 of 16 (50%) in the control group, 6 of 11 (54.5%) in the Foramulab/Dexa group and 9 of 12 (75%) in the Foralumab group. Among the 16 patients that remained symptomatic at the end of the study, anosmia (Domain 5) and cough (Domain 8) were the most common symptoms. There were anecdotal reports of rapid recovery from anosmia and ageusia in both Foralumab treated groups. Of note, our study was not designed to determine long-term effects of Foralumab on COVID-19 symptomatology.

**Table 3:** COVID-19 Symptomatology.

	Controlno. (%)		Foralumab/Dexano. (%)		Foralumabno. (%)			p-value*	
	Day -2	Day 13	Day -2	Day 13	Day -2	Day 13		Day-2	Day 13
**Domain 1	11 (68.8)	1 (6.2)	10 (90.9)	0 (0)	10 (83.3)		1 (8.3)	0.35	0.64
Domain 2	9 (56.2)	0 (0)	6 (54.5)	0 (0)	7 (58.3)		0 (0)	0.98	0.58
Domain 3	5 (31.2)	3 (18.8)	1 (9.1)	0 (0)	0 (0)		0 (0)	0.06	0.10
Domain 4	13 (81.2)	2 (12.5)	9 (81.8)	2 (18.2)	10 (83.3)		0 (0)	0.99	0.33
Domain 5	12 (75.0)	3 (18.8)	10 (90.9)	3 (27.3)	8 (66.7)		1 (8.3)	0.38	0.49
Domain 6	7 (43.8)	0 (0)	5 (45.5)	0 (0)	6 (50.0)		0 (0)	0.95	0.58
Domain 7	7 (43.8)	2 (12.5)	8 (72.7)	1 (9.1)	5 (41.7)		0 (0)	0.24	0.46
Domain 8	13 (81.2)	2 (12.5)	8 (72.7)	2 (18.2)	7 (58.3)		2 (16.7)	0.41	0.91
^†^Domains/pt. **^#^** (mean ± SD)	4.8 ( ± 1.5)	0.8 ( ± 1.0)	5.2 ( ± 0.6)	0.7 ( ± 0.9)	4.4 ( ± 1.3)		0.3 ( ± 0.7)	0.23	0.37

**For each Domain, the number (%) of patients experiencing symptoms in that Domain at day -2 and day 13 are shown. *p-value for each domain is from a chi-squared test comparing the proportions across the three groups at each time point. For the domains per patient, the three groups were compared using a Kruskal-Wallis test. **^#^** Plus–minus values are means ± SD. ^†^Domains/pt = the average number of Domains in which patients experienced symptoms. Pt, patient; Dexa, Dexamethasone.

### Adverse Events

Eleven patients (28%) experienced an adverse event including headache (n=4) burning in the nostril (n=1), retrosternal pain (n=2), pustular lesions and itching in cervical area (n=1), dysuria (n=1), tachycardia associated with anxiety (n=1) and insomnia (n=1) (**Table 4**). No serious adverse events were observed, and all patients completed the study. The incidence and severity of adverse events was determined according to the National Cancer Institute Common Terminology Criteria for Adverse Events, version 5.0.

**Table 4:** Adverse events.

		Control (n = 16)	Foralumab/Dexa (N = 11)	Foralumab (n = 12)
Headache	1		0	3
Burning in the nostril	0		0	1
Retrosternal pain	0		2	0
Pustular Lesions and Itching in cervical area	0		0	1
Dysuria	1		0	0
Tachycardia (anxiety)	0		1	0
Insomnia	0		0	1

The incidence and severity of adverse events was determined according to the National Cancer Institute Common Terminology Criteria for Adverse Events, version 5.0. Dexa= Dexamethasone.

### White Blood Cells

Lymphocyte, monocyte and neutrophil counts were obtained on days -2, 5 and 10 (**Table 5**). No reduction in white blood cells was observed with treatment. Lymphocytes and monocytes were increased in the Foralumab/Dexa cohort on day 5; p=0.002 and p=0.038, respectively but not on the last day on dosing. Lymphocytes were not elevated significantly above Baseline in the foralumab alone group or the control group. No differences in neutrophil counts were observed. In three group comparisons, a difference was observed in the control *vs*. the Foralumab/Dexa group and the Foralumab *vs*. Foralumab/Dexa for lymphocytes at day 5; p= 0.008 and p=0.027, respectively.

**Table 5:** White blood cell analysis.

	Day -2	Day 5	Day 10
Lymphocytes	Mean ( ± SD)^#^	Mean ( ± SD)	Mean ( ± SD)
Control	1819.9 ( ± 585.6)	2138.3 ( ± 649.6)	2091.5 ( ± 357.3)
Foralumab/Dexa	1770.8 ( ± 431.2)	3397.9 ( ± 1384.3)	2190.8 ( ± 666.2)
Foralumab	1722.6 ( ± 623.7)	2123.2 ( ± 754.9)	2135.2 ( ± 460.2)
*Three group comparison (p-value)	0.90	0.002	0.88
^†^Control - Foralumab/Dexa	–	0.008	0.79
^†^Control - Foralumab	–	0.87	0.82
^†^Foralumab - Foralumab/Dexa	–	0.027	0.74
**Monocytes**	Mean ( ± SD)	Mean ( ± SD)	Mean ( ± SD)
Control	514.8 ( ± 227.1)	634.2 ( ± 309.9)	573.8 ( ± 182.9)
Foralumab/Dexa	574.3 ( ± 344.1)	826.3 ( ± 380.5)	656 ( ± 339.9)
Foralumab	487.6 ( ± 180.2)	503.7 ( ± 112.2)	553.9 ( ± 124.9)
*Three group comparison (p-value)	0.70	0.038	0.52
^†^Control - Foralumab/Dexa	–	0.31	0.79
^†^Control - Foralumab	–	0.39	0.90
^†^Foralumab - Foralumab/Dexa	–	0.05	0.60
**Neutrophils**	Mean ( ± SD)	Mean ( ± SD)	Mean ( ± SD)
Control	3236.4 ( ± 1725.8)	4816.4 ( ± 2169.2)	4274.9 ( ± 1596.2)
Foralumab/Dexa	4087.2 ( ± 2785)	5160.6 ( ± 2633.5)	4197.8 ( ± 1794)
Foralumab	3138.4 ( ± 1734.9)	3537.8 ( ± 1070.3)	4308.6 ( ± 1629.7)
*Three group comparison (p-value)	0.48	0.14	0.98
^†^Control - Foralumab/Dexa	–	0.64	0.54
^†^Control - Foralumab	–	0.24	0.71
^†^Foralumab - Foralumab/Dexa	–	0.44	0.44

**^#^** Plus–minus values are means ± SD. values are/mm^3^.

*P-value for three group comparison of change from baseline (day -2) is provided.

^†^P-values of difference between treatment groups in change from baseline from linear mixed model. Dexa, Dexamethasone.

### Inflammatory Biomarkers

**Figure 2:**
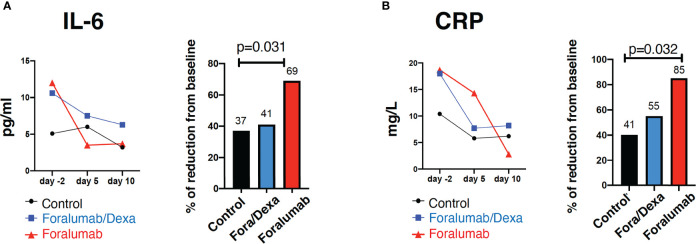
Blood inflammatory markers IL-6 and C-reactive protein. Serum quantification and percentage of reduction of **(A)** IL-6 and **(B)** C-reactive protein. Linear regression was used to compare three-group comparison of each time point. A linear mixed model with a random intercept was used to baseline (day -2) comparison. Percent of baseline was compared using Wilcoxon rank sum test. p= <0.05. IL, Interleukin, CRP, c-reactive protein, Dexa, dexamethasone.

We quantified serum levels of IL-6, CRP and D-dimer on days -2, 5 and 10. IL-6 and CRP levels may be linked to worse outcome in COVID-19 (32, 33) and anti-IL-6 therapy is being investigated as immunotherapy in COVID (34–36). As shown in **Figure 2** Foralumab resulted in a 69% reduction in IL-6 levels at day 10 (p=0.031) and 85% reduction in CRP at day 10 (p=0.032).

As shown in **Table 6**, three group comparisons showed a difference between Control *vs* Foralumab at a day 5 (p=0.01) and at day 10 (p=0.031) and in CRP on day 10 (p=0.032). No significant differences were observed in D-dimer serum levels among groups. We also measured serum IL-18. Paired analysis showed that IL-18 in the Foralumab group was 46.1 (± 15.5) pg/ml before treatment and 37.6 (± 12.6) pg/ml after treatment (p=0.054). There were no significant changes in serum IL-18 following treatment in the Foralumab/DEXA (p=0.16) or Control groups (p=0.43).

**Table 6:** Blood inflammatory biomarkers.

Biomarker	Day -2	Day 5	Day 10
IL-6	(Mean ± SD)^#^	(Mean ± SD)	(Mean ± SD)
Control	5.1 ( ± 5.4)	6 ( ± 7.5)	3.2 ( ± 1.9)
Foralumab/Dexa	10.6 ( ± 9.2)	7.5 ( ± 9.6)	6.3 ( ± 9.2)
Foralumab	12 ( ± 11.5)	3.5 ( ± 3)	3.7 ( ± 2.4)
Three group comparison (p-value)	0.09	0.41	0.29
Control - Foralumab/Dexa	–	0.20	0.40
^†^Control - Foralumab	–	0.01	0.031
^†^Foralumab - Foralumab/Dexa	–	0.42	0.32
**CRP**	(Mean ± SD)	(Mean ± SD)	(Mean ± SD)
Control	10.4 ( ± 19.6)	5.8 ( ± 12.8)	6.2 ( ± 12.2)
Foralumab/Dexa	18 ( ± 25.7)	7.7 ( ± 22.1)	8.2 ( ± 15.3)
Foralumab	18.7 ( ± 24.5)	14.3 ( ± 18.7)	2.8 ( ± 3.6)
Three group comparison (p-value)	0.56	0.44	0.52
^†^Control - Foralumab/Dexa	–	0.27	0.57
^†^Control - Foralumab	–	0.87	0.032
^†^Foralumab - Foralumab/Dexa	–	0.23	0.26
**D-dimer**	(Mean ± SD)	(Mean ± SD)	(Mean ± SD)
Control	368.8 ( ± 156.7)	888.6 ( ± 1635.6)	573.5 ( ± 436.8)
Foralumab/Dexa	434.6 ( ± 216.8)	555.5 ( ± 376.6)	417.1 ( ± 210.4)
Foralumab	499.5 ( ± 317.3)	757.7 ( ± 872.5)	551.8 ( ± 356.5)
Three group comparison (p-value)	0.34	0.77	0.52
^†^Control - Foralumab/Dexa	–	0.34	0.35
^†^Control - Foralumab	–	0.28	0.64
^†^Foralumab - Foralumab/Dexa	–	0.56	0.92

**^#^** Plus–minus values are means ± SD.

*P-value for three group comparison of change from baseline (day -2) is provided.

^†^P-values of difference between treatment groups in change from baseline from linear mixed model.

Interleukin, CRP, c-reactive protein, Dexa, dexamethasone.

IL-6 (pg/ml); CRP (mg/L); D-dimer (ng/ml FEU).

### Lung CT Analysis

**Figure 3:**
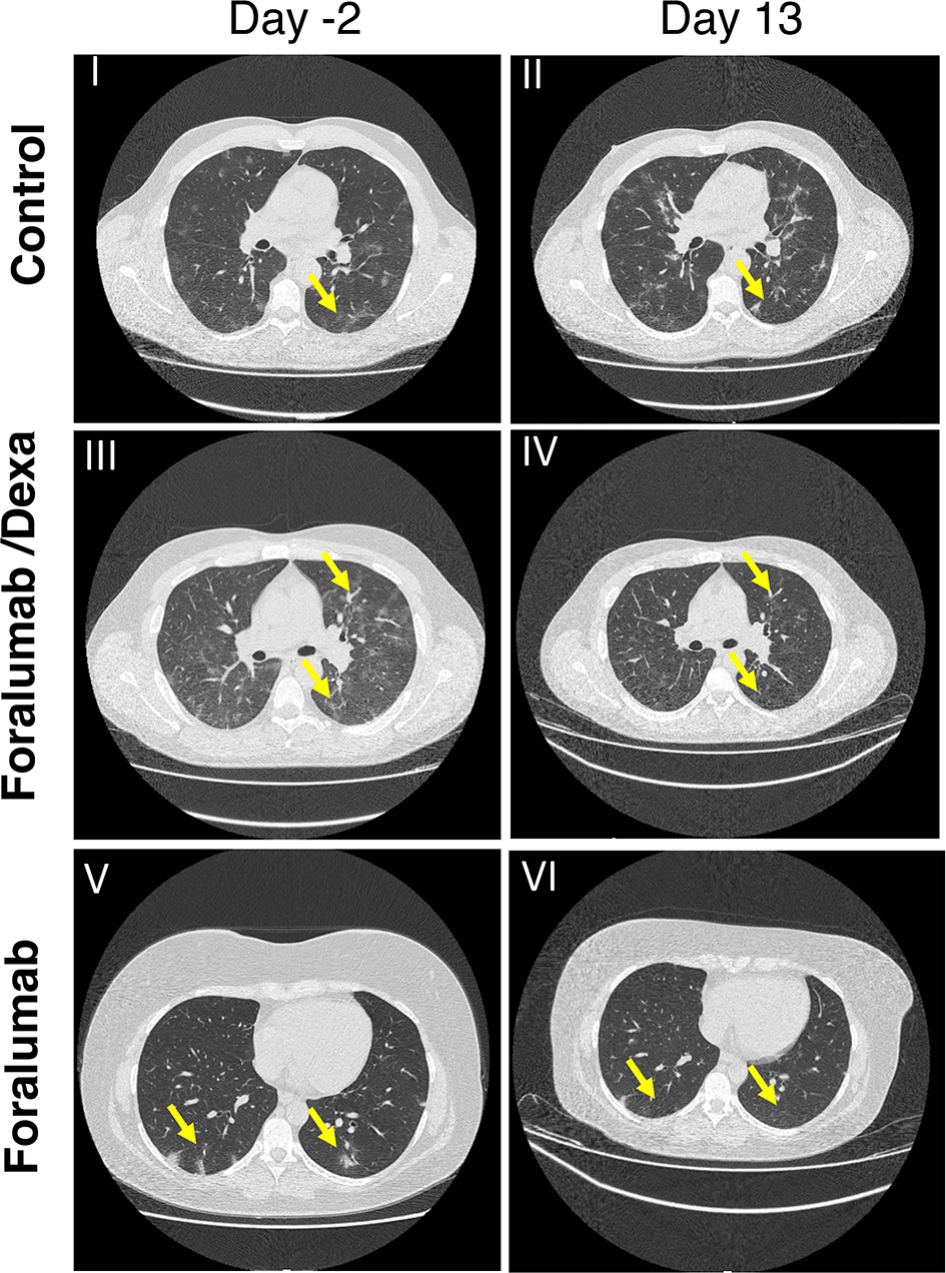
Lung CT Scan: CT chest in COVID-19 patients by treatment group. I-II: Axial images in a control patient shows widespread ground glass opacity (anterior and posterior segments of bilateral upper and right middle lobes) two days prior to treatment (I) showing significant progression at 13 days follow up (II). III-IV: Axial images in a patient treated with Foralumab/Dexa showing both widespread ground glass opacity in the anterior and posterior segments and consolidation in both lower lobes (III) demonstrating partial resolution on the 13 follow up day scan (IV). V-VI: Axial images in a patient who received Foralumab showing ground glass opacity of posterior segments of lungs (V) demonstrating interval resolution on 13 follow up day scan (VI). Dexa, dexamethasone.

Computerized tomography (CT) of the lung was obtained prior to treatment at day -2 and at study completion on day 13 and analyzed as described in the methods. Lung CT scan was not obtained in 2 control subjects and 1 Foralumab/Dexa subject. Each patient was classified as worse, stable, improved or markedly improved. As shown in **Table 7**, 1/10 of the Foralumab/Dexa subjects worsened. 10/14 control subjects, 2/10 Foralumab/Dexa subjects and 2/12 Foralumab subjects remained stable. Regarding improvement, because 6 patients in the control group and 2 in the Foralumab/Dexa group had no lung involvement on day -2, they were not able to improve. Thus, improvement occurred in 3/8 control, 1/8 Foralumab/Dexa and 5/12 in the Foralumab group. Marked improvement was observed in 1/8 control, 6/8 in the Foralumab/Dexa and 5/12 in the Foralumab group. Thus, marked improvement was predominantly observed in subjects receiving Foralumab/Dexa or Foralumab alone. Control *vs*. Foralumab/Dexa, p=0.01 and Control *vs*. Forlumab/Dexa+Foralumab, p=0.04 (chi-square analysis) (**Figure 3**).

**Table 7:** Lung CT scan analysis.

	Control		Foralumab/Dexa	Foralumab
Worsened	0/14	1/10		0/12
Stable	10/14	2/10		2/12
Improved	3/8	1/8		5/12
Marked Improvement*	1/8	6/8		5/12

Patients were classified as worsened if they increased by one or more stage, improved if they decreased by one stage and as having marked improvement if they decreased by 2 or more stages.

Two patients in the control group and 1 in Foralumab/Dexa group did not undergo lung CT at day 13 and thus are not part of the analysis.

Six patients in the control group and 2 in the Foralumab/Dexa group had no lung involvement on day -2 and thus were not able to improve. They are not counted in the improved categories.

*p=0.01, Control vs. Foralumab/Dexa and p=0.04, Control vs. Forlumab/Dexa+Foralumab, chi-square analysis.

## Discussion

Intravenous anti-CD3 monoclonal antibody has been used to treat graft rejection (37) and is being investigated in type 1 diabetes (38). Intravenous anti-CD3 modulates CD3 from the T cell surface and causes a reduction of lymphocytes whereas mucosal (nasal and oral) anti-CD3 acts by inducing regulatory T cells at the mucosal surface that then act systemically (39). In animal studies no reduction in lymphocytes has been observed following nasal anti-CD3 (40). Consistent with this, no reduction in lymphocyte counts was observed in COVID-19 patients treated with 100μg nasal anti-CD3 for 10 days or in healthy volunteers treated with 250μg Foralumab for 5 consecutive days (unpublished). In our study, no significant adverse events were observed and treatment with Foralumab was generally well tolerated.

As this was an open label exploratory clinical study, it was not specifically powered for statistical analysis and the major limitations of the study are its small size and the over the counter use of corticosteroids.

Systemic corticosteroids are commonly used to treat COVID-19 patients who are hospitalized and have decreased oxygen saturation and international guidelines recommend moderate doses of dexamethasone for a short period of time when hemodynamic parameters are compromised (7, 41). The use of corticosteroid treatment for noncritically ill is controversial (42).

In this pilot trial, we tested the effect of Foralumab alone *vs*. Foralumab given with a three-day course of dexamethasone. Foralumab/Dexa appeared to be more effective in improving lung inflammation than Foralumab alone. Foralumab alone was more effective than Foralumab/Dexa and controls in decreasing inflammatory blood markers at day 10 as measured in a three group comparison.

Given the anti-inflammatory effects of Foralumab and the positive safety profile of Foralumab treated COVID-19 patients, further studies are warranted. Of particular interest would be the degree to which nasal Foralumab might benefit hospitalized subjects with more severe disease. In addition, since it can easily be administered as an outpatient, it could be used on non-hospitalized subjects with COVID-19 and may be of benefit to speed recovery and prevent disease worsening and hospitalization. Because the anti-inflammatory effect of the nasally administered Foralumab is through the modulation of the immune system and not by directly targeting COVID-19, if effective, it would be expected to be useful for newly identified COVID-19 variants and could be given in combination with other drugs.

Immunomodulatory agents that enhance regulatory immune responses are believed to play an important role in modulating disease in patients with COVID-19 by suppressing hyperreactive immune responses (12–14, 43). In animals, we have shown that nasal anti-CD3 modulates the immune response by inducing IL-10-producing Tregs (26, 29) without the occurrence of potential adverse events associated with parenteral anti-CD3 therapy (39). Although we hypothesize that the effect of nasal Foralumab in COVID-19 patients is related to the induction of Tregs, Treg function was not measured in the current study.

In summary, although we found positive effects of nasal Foralumab as measured by decreases in IL-6 and CRP and improvement on lung CT scans in mild to moderate COVID-19 patients, our results must be taken with caution and we cannot conclude that nasal Foralumab has a beneficial effect in COVID-19 until larger studies are performed. Nonetheless, our results have identified a novel immunomodulatory therapy that potentially could have a significant benefit in patients suffering from COVID-19.

## Data Availability Statement

The original contributions presented in the study are included in the article/supplementary material. Further inquiries can be directed to the corresponding authors.

## Ethics Statement

The studies involving human participants were reviewed and approved by Universidade Metropolitana de Santos – UNIMES (CAAE: 38056120.1.0000.5509). The patients/participants provided their written informed consent to participate in this study.

## Author Contributions

TM designed the study, led and monitored this clinical study, and wrote the manuscript. KM designed the study, clinically monitored the study, and performed Lung CT analysis. GD and TS recruited patients, performed clinical exams, registered drug intake and medical past history. RGD, monitored the study and helped collect BMI data. FP and AC supported patient recruitment. MS performed biomarkers assays. CMB-A participated in drug development. GK performed serum and plasma collection. JJ and VP performed drug stability assays. SI and KC performed Lung CT scan analysis. BH performed statistical analysis. RR designed the study and reviewed manuscript. RAD helped supervise the study at Santa Casa de Santos Hospital. KS helped design the study and reviewed the manuscript. HW designed the study and helped write the manuscript. All authors contributed to the article and approved the submitted version.

## Conflict of Interest

JJ, VP and KS are employees of Tiziana, Life Sciences. HW is chair of the Scientific Advisory Board of Tiziana and received consulting fees and stock options from the company. TM and KM received consulting fees from the company.

The remaining authors declare that the research was conducted in the absence of any commercial or financial relationships that could be construed as a potential conflict of interest.

## Publisher’s Note

All claims expressed in this article are solely those of the authors and do not necessarily represent those of their affiliated organizations, or those of the publisher, the editors and the reviewers. Any product that may be evaluated in this article, or claim that may be made by its manufacturer, is not guaranteed or endorsed by the publisher.
