# Corrigendum: Nasal Administration of Anti-CD3 Monoclonal Antibody (Foralumab) Reduces Lung Inflammation and Blood Inflammatory Biomarkers in Mild to Moderate COVID-19 Patients: A Pilot Study

**DOI:** 10.3389/fimmu.2021.815812

**Published:** 2022-01-12

**Authors:** Thais G. Moreira, Kimble T. F. Matos, Giovana S. De Paula, Thais M. M. Santana, Raquel G. Da Mata, Fernando C. Pansera, Andre S. Cortina, Marcelle G. Spinola, Clare M. Baecher-Allan, Gerson D. Keppeke, Jules Jacob, Vaseem Palejwala, Karen Chen, Saef Izzy, Brian C. Healey, Rafael M. Rezende, Rogerio A. Dedivitis, Kunwar Shailubhai, Howard L. Weiner

**Affiliations:** ^1^ Ann Romney Center for Neurologic Diseases, Department of Neurology, Brigham and Women’s Hospital, Harvard Medical School, Boston, MA, United States; ^2^ Escola Paulista de Medicina, Universidade Federal de São Paulo, São Paulo, Brazil; ^3^ Santa Casa de Misericordia de Santos, Santos, Brazil; ^4^ Tiziana LifeScience, Doylestown, PA, United States; ^5^ Department of Radiology, Boston Children’s Hospital, Harvard Medical School, Boston, MA, United States

**Keywords:** foralumab, anti-CD3, COVID-19, SARS-CoV-2, immune responses

**Clare M. Baecher-Allan** was not included as an author in the published article. The corrected Author Contributions Statement appears below.

The authors apologize for this error and state that this does not change the scientific conclusions of the article in any way. The original article has been updated.

“TM designed the study, led and monitored this clinical study, and wrote the manuscript. KM designed the study, clinically monitored the study, and performed Lung CT analysis. GD and TS recruited patients, performed clinical exams, registered drug intake and medical past history. RGD, monitored the study and helped collect BMI data. FP and AC supported patient recruitment. MS performed biomarkers assays. **CMB-A participated in drug development**. GK performed serum and plasma collection. JJ and VP performed drug stability assays. SI and KC performed Lung CT scan analysis. BH performed statistical analysis. RR designed the study and reviewed manuscript. RAD helped supervise the study at Santa Casa de Santos Hospital. KS helped design the study and reviewed the manuscript. HW designed the study and helped write the manuscript. All authors contributed to the article and approved the submitted version.”

## Publisher’s Note

All claims expressed in this article are solely those of the authors and do not necessarily represent those of their affiliated organizations, or those of the publisher, the editors and the reviewers. Any product that may be evaluated in this article, or claim that may be made by its manufacturer, is not guaranteed or endorsed by the publisher.

